# Influence of Different Indole-3-Butyric Acid (IBA) Doses, *Aloe vera* Gel, and Rooting Media on Rooting Performance and Growth of Tea (*Camellia sinensis*) Cuttings

**DOI:** 10.3390/plants15142155

**Published:** 2026-07-13

**Authors:** Turan Yüksek, Açelya Şenal, Filiz Yüksek, Çağla Sağır, Servet Çeltek, Türker Oğuztürk

**Affiliations:** 1 Department of Landscape Architecture, Faculty of Engineering and Architecture, Recep Tayyip Erdoğan University, Rize 53020, Türkiye; turan.yuksek@erdogan.edu.tr (T.Y.); acelya_senal19@erdogan.edu.tr (A.Ş.); cagla_sagir24@erdogan.edu.tr (Ç.S.); servwetxcx@icloud.com (S.Ç.); 2 Rize Forest Management Directorate, Trabzon Regional Forestry Directorate, General Directorate of Forestry, Ministry of Agriculture and Forestry, Rize 53020, Türkiye; filiz6108@gmail.com

**Keywords:** natural biostimulant, auxin response, nursery propagation, plantlet establishment, tea

## Abstract

This study evaluated the effects of different indole-3-butyric acid (IBA) doses, *Aloe vera* gel, and rooting media on the rooting performance and subsequent growth of vegetative tea (*Camellia sinensis*) cuttings. Cuttings collected during the second flush period were subjected to seven IBA concentrations (2000–8000 ppm), *Aloe vera* gel, or no pre-treatment and rooted in perlite or clay soil under greenhouse conditions using a randomized complete block design with three replications. Rooting and growth parameters were monitored for 120 days, followed by a 15-week growth assessment after transplanting. The rooting medium significantly influenced root length and cutting length, with clay soil promoting greater root elongation and perlite enhancing shoot growth. Among IBA treatments applied in perlite, intermediate concentrations (4000–6000 ppm) resulted in comparatively favorable rooting performance and growth compared to lower or higher doses. The highest rooting and survival rates were obtained with 5000 ppm IBA combined with perlite, while the greatest root number was observed at 6000 ppm IBA. *Aloe vera* gel was associated with greater root length particularly in soil-based media, suggesting its potential as a natural rooting stimulant under the tested soil-based conditions. Overall, the results indicate that rooting success and early growth of tea cuttings were influenced by both the auxin concentration and rooting medium under the tested greenhouse conditions. Under the conditions of the present study, the use of intermediate IBA doses in perlite appears to be an effective propagation approach, while *Aloe vera* gel may represent a promising alternative for soil-based rooting systems. These findings contribute to the optimization of vegetative propagation protocols for tea and support the production of high-quality, cost-effective planting material.

## 1. Introduction

Tea (*Camellia sinensis* (L.) Kuntze) is one of the world’s most economically and socially important agricultural crops, cultivated across approximately 5.3 million hectares globally [1]. China has the largest tea cultivation area, while Türkiye represents an important tea-producing country, particularly when unregistered plantations are taken into account. The global tea sector provides employment for nearly 13 million people [2], and the tea market is projected to reach a value of USD 98.29 billion by 2033 [3]. Along with the continued expansion of tea cultivation and consumption worldwide, Türkiye ranks first in global per capita tea consumption, with an annual consumption of approximately 4 kg per person [4]. As the second most consumed beverage after water, tea plays a vital role in socio-cultural life while also supporting rural livelihoods and sustainable agricultural development.

Despite the economic importance of tea production in Türkiye, nursery propagation and vegetative plantlet production practices are still largely based on traditional approaches. One of the major constraints in establishing or renewing tea plantations is the production of vigorous, high-quality, and affordable plantlets for sustainable cultivation. Plantlets produced without appropriate vegetative propagation techniques often exhibit poor rooting ability, low survival rates, and weak early growth performance. In addition, studies on tea propagation under Turkish conditions remain relatively limited. Previous research has mainly focused on the effects of different Indole-3-Butyric Acid (IBA) doses in perlite media [5,6,7,8], greenhouse propagation without hormone treatments [9], and rhizobacteria-assisted rooting applications [10]. However, the combined effects of natural biostimulants, rooting media, and pre-treatment applications on both rooting success and subsequent plantlet development in tea cuttings have not yet been sufficiently investigated.

Successful root initiation is a key determinant of vegetative propagation efficiency in many horticultural and agricultural plant species. Root formation in cuttings is regulated by complex physiological mechanisms involving auxin balance, cell division, carbohydrate metabolism, water relations, and antioxidant defense systems [11,12]. Consequently, increasing attention has been directed toward natural biostimulants as sustainable alternatives or supplements to synthetic rooting hormones [13,14]. In this context, recent studies have also shown that organic waste-derived materials, such as vermicompost and liquid vermicompost, can support sustainable plant production and improve early developmental responses in *Camellia sinensis*, indicating the broader potential of natural and organic inputs in tea propagation systems [15,16]. Among these, *Aloe vera* gel has gained considerable interest because of its rich composition of bioactive compounds, including polysaccharides, phenolics, vitamins, enzymes, and antioxidant substances [16,17,18]. These compounds are reported to stimulate cell division, promote wound-induced tissue formation and callus development, alleviate oxidative stress, and contribute to tissue water balance [19,20,21]. Furthermore, *Aloe vera* extracts may exhibit auxin-like activity and support endogenous plant growth regulators involved in root initiation processes [22,23,24]. Based on these reported properties, *Aloe vera* gel may act as a natural biostimulant that supports adventitious root formation through combined effects on cellular activity, antioxidant protection, tissue hydration, and hormonal balance. Taken together, these properties indicate that *Aloe vera* gel may have potential as a natural biostimulant for improving rooting performance and early plantlet development in tea cuttings.

Although rooting capacity is a key criterion in vegetative propagation, the successful establishment of rooted cuttings under field conditions also depends on vigorous shoot development and overall plantlet quality. Plantlets with improved shoot growth and plant vigor generally show better adaptation after transplantation, higher survival rates, and lower maintenance requirements. Despite the importance of post-rooting growth performance, the effects of different rooting treatments on subsequent shoot development and early plantlet growth in tea cuttings remain poorly understood. In particular, limited information is available regarding the combined influence of *Aloe vera* applications, rooting media, and pre-treatment practices on both root and shoot development under greenhouse propagation conditions.

The objectives of this study were to: (i) evaluate the effects of *Aloe vera* gel and different rooting media on the rooting performance of tea cuttings collected during the second flush period; (ii) determine the influence of different IBA doses on root and shoot development; and (iii) assess the effects of different treatment combinations on rooting success, survival, and early plantlet growth. Accordingly, the study tested the hypotheses that rooting media, pre-treatments, and IBA applications significantly influence root development, shoot growth, and overall propagation performance of tea cuttings. The study was designed as an applied nursery-scale propagation experiment rather than a physiological, biochemical, or molecular research study; therefore, the results are interpreted within the specific genotype, cutting stage, rooting media, and greenhouse conditions tested.

To the best of our knowledge, limited information is available on the combined evaluation of *Aloe vera* gel, rooting media, and varying IBA doses in relation to both rooting and early plantlet growth of tea cuttings under greenhouse conditions. The study contributes to the existing literature by evaluating not only rooting success and survival rates, but also early plantlet growth responses following different treatment combinations. In addition, the temporal growth responses of rooted cuttings were monitored throughout the propagation period, providing a more comprehensive evaluation of post-rooting development and plantlet performance in tea propagation.

## 2. Materials and Methods

### 2.1. Study Area and Land Use History

Tea cuttings used in this study were collected from a tea plantation located in Yemişli Village, Pazar district, Türkiye (41°08′46.08″–41°08′47.79″ N, 40°55′09.67″–40°55′10.93″ E). The plantation is situated at an average elevation of 185 ± 3 m above sea level with a south-southeast aspect [25]. The soils of the study area are clay-textured and developed over agglomerate parent material [26]. The region is characterized by a humid oceanic climate, with an annual mean temperature of 13.57 °C and an average annual precipitation of 2032 mm. According to the Thornthwaite climate classification, the area is classified as mesothermal and very humid (A B’1 r a’) [27]. The tea plantation was established from seed in 1975. Rejuvenation pruning was conducted approximately every 12 years at a height of nearly 15 cm above ground level, followed by the application of approximately 700 kg da^−1^ of farmyard manure. In addition, compound NPK fertilizer was applied annually at a rate of 120 kg da^−1^ between 1990 and 2024. However, apart from routine fertilization practices, no intensive cultural management or additional rejuvenation treatments were implemented during this period.

### 2.2. Collection of Plant Material

The vegetative tea cuttings used in the experiments were collected from healthy and vigorously growing tea bushes during the second flush period on 4 August 2024. Current-season semi-hardwood shoots showing no visible symptoms of disease, pest infestation, or mechanical damage were selected as source material. Uniform cuttings approximately 12–15 cm in length, each containing 3–4 leaves and at least two nodes, were prepared to minimize variation among treatments. Immediately after collection, the cuttings were wrapped in moist cloths to prevent moisture loss and transported in a carrying box to the Research and Application Greenhouse of the Department of Landscape Architecture at Recep Tayyip Erdoğan University for subsequent treatment and planting.

### 2.3. Pretreatment of Cuttings and Application of IBA and Aloe vera Gel

The vegetative tea cuttings brought from the study area were trimmed to 10 cm in length, each retaining three half-leaves. The basal ends of the cuttings were cut at a 45° angle. IBA (Erhan Gül Zirai İlaç Tohum ve Gübre Toptan Ticaret, Antalya, Türkiye) solutions were prepared freshly before application. The required amount of indole-3-butyric acid powder was first dissolved in a small volume of 95% ethanol (Kanmer Mühendislik, Trabzon, Türkiye) to ensure complete dissolution and then brought to the final volume with distilled water (laboratory-prepared distilled water) to obtain the target concentrations of 2000, 3000, 4000, 5000, 6000, 7000, and 8000 ppm. The basal 1–2 cm portion of each cutting was dipped into the corresponding IBA solution for 5 s before planting. The cuttings were dipped for 5 s into different IBA solutions (2000 ppm, 3000 ppm, 4000 ppm, 5000 ppm, 6000 ppm, 7000 ppm, and 8000 ppm) or into *Aloe vera* gel, and then planted in the pre-prepared rooting media.

Healthy *Aloe vera* (L.) Burm.f. plants approximately two years old and cultivated under the same greenhouse conditions at the RTEU Research and Application Greenhouse were used as the source of *Aloe vera* gel. On 5 August 2024, fully developed, healthy green leaves showing no visible symptoms of disease, pest infestation, or mechanical damage were harvested from the outer portion of the plants. The leaves were washed thoroughly with tap water and manually peeled to remove the outer epidermal tissue. The transparent inner gel was then collected, pooled, and homogenized to obtain a uniform gel preparation. The freshly prepared gel was used immediately as 100% *Aloe vera* gel without dilution. No commercial additives, preservatives, solvents, or other substances were added during gel preparation. To minimize potential changes in biological activity, the gel was not stored and was applied on the same day it was prepared. For the *Aloe vera* treatment, the basal 1–2 cm portion of each cutting was completely immersed in the freshly homogenized gel for approximately 5 s, following the same short-dip procedure used for the IBA treatments. Immediately after treatment, the cuttings were planted in the designated rooting media. The short-dip method was adopted to ensure consistency among pre-treatment applications and to avoid excessive gel accumulation around the cutting base prior to planting.

### 2.4. Experimental Design

In the experiments, two different rooting media (perlite and clay soil), seven different IBA doses, and fresh *Aloe vera* gel (100%) were used (Table 1). IBA treatments were applied only in perlite (Kanmer Mühendislik, Trabzon, Türkiye) to minimize variability arising from soil heterogeneity and to focus on dose-dependent auxin responses under controlled rooting conditions. Therefore, the present experimental design was not structured as a full factorial trial including all IBA concentrations across both rooting media. The experimental structure consisted of two complementary comparisons rather than a full factorial design. First, dose-dependent IBA responses were evaluated in perlite, which was selected as a relatively inert and homogeneous rooting medium. Second, the effects of rooting medium and *Aloe vera* gel were evaluated using control and *Aloe vera*-treated cuttings in both perlite and clay soil. Accordingly, the results related to IBA concentration should be interpreted primarily within the perlite rooting medium, whereas comparisons involving *Aloe vera* gel and untreated controls were used to evaluate the influence of rooting media under the tested pre-treatment conditions.

The experiment was conducted in rooting beds located in the Research and Application Greenhouse of the Department of Landscape Architecture at Recep Tayyip Erdoğan University. The rooting beds were 100 cm wide, 200 cm long, and 30 cm deep, and were filled with the respective rooting media. During the rooting period, no supplemental heating, cooling, or artificial lighting was used. Therefore, environmental conditions inside the greenhouse largely reflected the prevailing climatic conditions of the region. According to data obtained from the nearest meteorological station, monthly mean air temperatures during the experimental period (August–November 2024) ranged from 11.6 to 26.0 °C, while monthly mean relative humidity ranged from 74.1% to 81.3%. Natural sunlight was used throughout the experiment.

**Figure 1:**
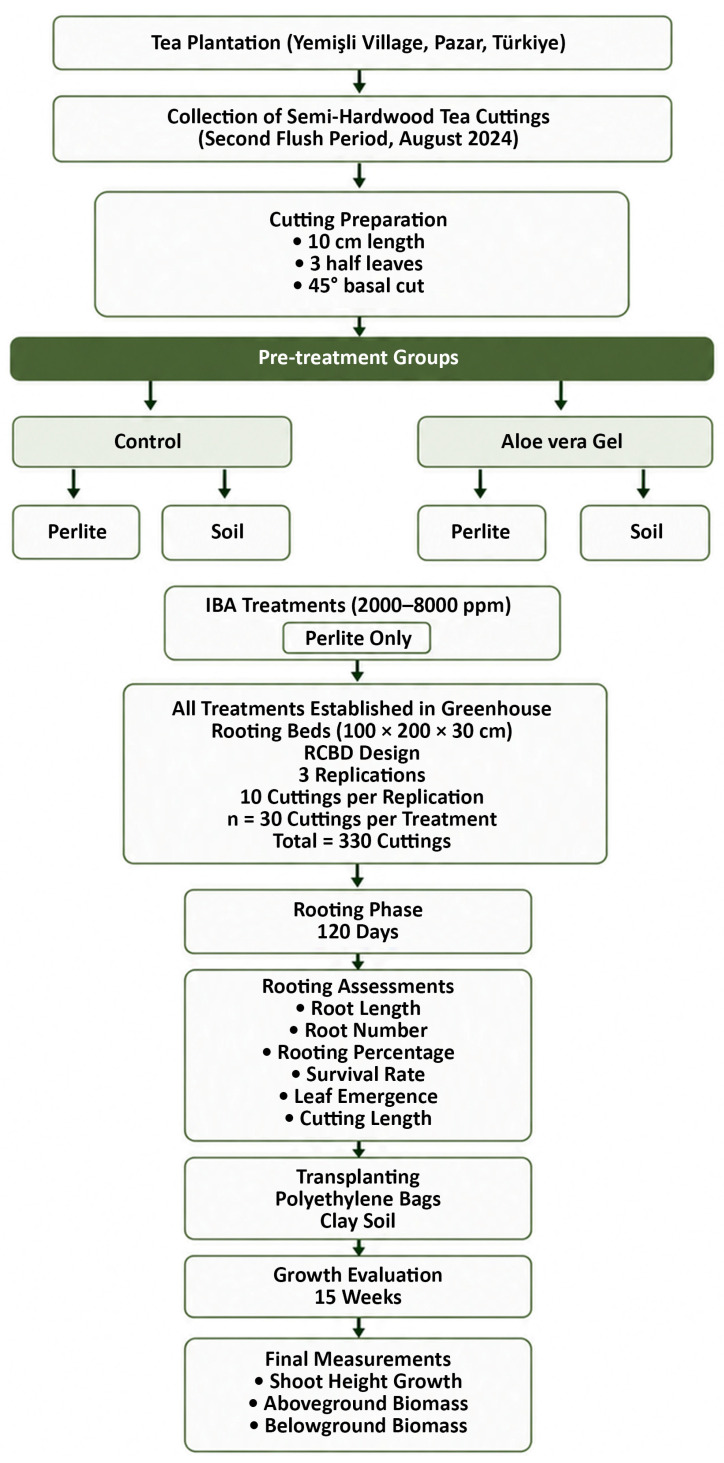
Experimental workflow of tea cutting propagation study.

The experimental design was established according to the randomized complete block design method with three replications. In each replication, 10 vegetative cuttings were planted at 5 cm intervals in the two different rooting media. The vegetative cuttings in the rooting media were irrigated using a misting system three times a week with a total of 161.2 L/m^2^ of tap water. The height of the vegetative cuttings in the rooting media was measured every two weeks over a period of 120 days. The 120-day evaluation period was selected to assess not only initial root formation but also rooting stability, survival, and early cutting development before transplanting. At the end of 120 days, rooted tea plantlets obtained under different pre-treatments and rooting media were transferred to clay soil. The development of tea plantlets transferred to polyethylene bags was monitored for 15 weeks, and some plant characteristics were measured and recorded. At the end of 15 weeks, three tea plantlets were randomly selected from each treatment. After washing off the soil, they were separated into underground and aboveground parts, and their weights were determined by weighing. The overall experimental workflow, including cutting collection, pretreatment applications, rooting phase, transplanting, and subsequent growth evaluation procedures, is summarized in Figure 1.

### 2.5. Measurements Carried out on Vegetative Tea Cuttings

Some plant parameters of vegetative cuttings and rooted plantlets in the experimental plots, including plantlet height, root collar diameter, root length, leaf number, root number, and flower bud number, were measured once every two weeks over a period of 120 days (Table 2).

### 2.6. Statistical Analysis

The effects of rooting medium (perlite and clay soil), pre-treatment (control and *Aloe vera*), and their interaction on root length, root number, and cutting length were analyzed using two-way ANOVA. This analysis was limited to control and *Aloe vera* treatments, which were applied in both rooting media. The effects of different treatment combinations on survival rate, rooting percentage, leaf emergence rate, root length, root number, and cutting length were evaluated using one-way ANOVA. Post hoc comparisons were performed using Duncan’s multiple range test at *p* < 0.05. Shoot height growth of rooted cuttings was recorded at two-week intervals over a 15-week period. Differences among treatment combinations at each observation time were analyzed using one-way ANOVA. All statistical analyses were performed using IBM SPSS Statistics (IBM Corp., Armonk, NY, USA) for Windows, Version 25.0 (IBM Corp., Armonk, NY, USA).

## 3. Results

### 3.1. Effects of Rooting Medium and Pre-Treatment on Rooting Characteristics

**Figure 2:**
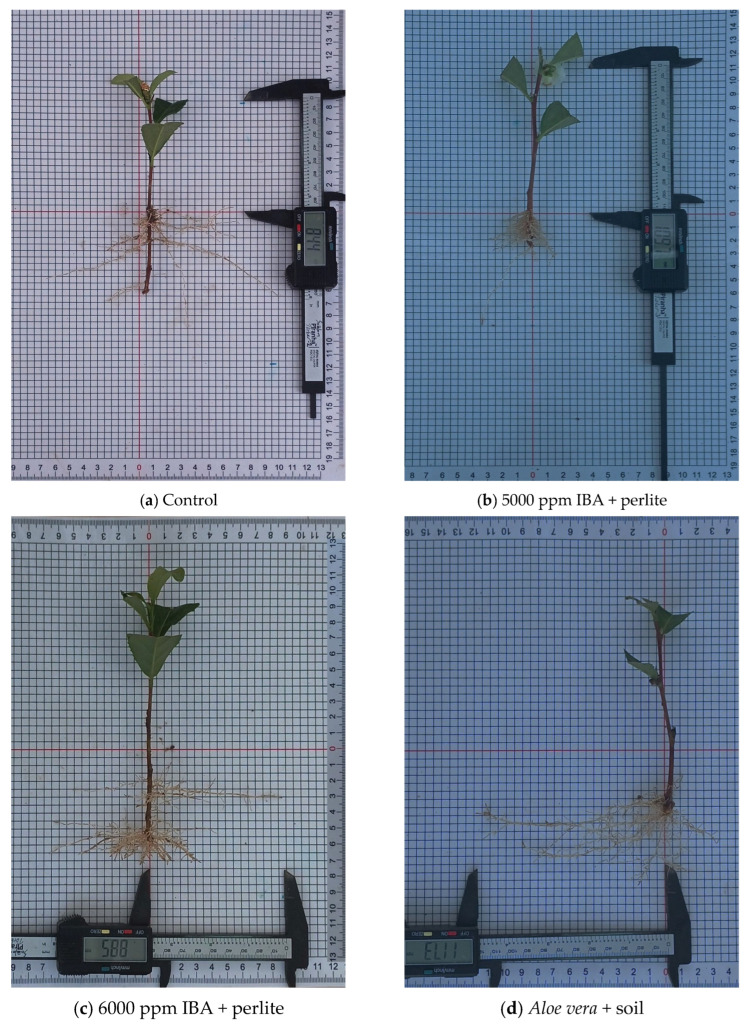
Representative rooted tea (*Camellia sinensis*) cuttings after 120 days under selected treatment combinations: (**a**) untreated control, (**b**) 5000 ppm IBA + perlite, (**c**) 6000 ppm IBA + perlite, and (**d**) *Aloe vera* gel + soil. The images visually illustrate representative treatment-related differences in root development and vegetative growth among selected treatments.

The effects of rooting medium, pre-treatment (control and *Aloe vera*), and their interaction on root length, root number, and cutting length are presented in Table 3. Root length and cutting length differed significantly between rooting media (*p* < 0.001), whereas no significant difference was observed for root number (*p* = 0.327). Cuttings rooted in clay soil exhibited greater root length compared to those rooted in perlite, while cuttings grown in perlite showed significantly greater cutting length. Pre-treatment with *Aloe vera* did not result in statistically significant differences in root length, root number, or cutting length compared to untreated cuttings. Similarly, the interaction between the rooting medium and pre-treatment was not statistically significant for any of the evaluated parameters. Representative images of rooted tea cuttings obtained under selected treatments are shown in Figure 2.

### 3.2. Effects of Treatment Combinations on Survival, Rooting, and Leaf Emergence

The survival rate, rooting percentage, and leaf emergence rate of tea cuttings under different treatment combinations after 120 days are shown in Table 4. Survival rates formed two statistically distinct groups, rooting rates formed four groups, and leaf emergence rates formed three groups. The highest survival and rooting percentages were observed in cuttings treated with 5000 ppm IBA in perlite, followed by 6000 ppm IBA + perlite and 4000 ppm IBA + perlite treatments. In contrast, untreated control treatments exhibited lower survival and rooting rates. The leaf emergence rate was also highest in the 5000 ppm IBA + perlite treatment, while the lowest values were recorded in untreated cuttings rooted in perlite. Among *Aloe vera* treatments, *Aloe vera* + soil resulted in a higher leaf emergence rate compared to *Aloe vera* + perlite.

### 3.3. Effects of Treatment Combinations on Root and Shoot Growth

The effects of different pre-treatment and rooting medium combinations on root length, root number, and vegetative cutting length are summarized in Table 5. Treatment combinations significantly affected root length, root number, and cutting length (*p* < 0.001). The greatest root length was observed in cuttings treated with *Aloe vera* and rooted in soil, whereas the shortest root length occurred in untreated cuttings rooted in perlite. Among IBA treatments in perlite, 6000 ppm IBA resulted in the longest roots, while intermediate IBA doses (4000–5000 ppm) produced comparatively favorable root and shoot responses under the tested conditions.

### 3.4. Shoot Height Growth After Transplanting

Shoot height growth of rooted cuttings over a 15-week period following transplanting is presented in Table 6. Significant differences among treatment combinations were detected at all observation times (*p* < 0.05). In the early growth stages, cuttings rooted in perlite exhibited greater shoot elongation compared to those rooted in soil. Over time, treatment combinations involving intermediate IBA doses, particularly 4000 ppm IBA + perlite, consistently produced greater shoot height growth than other treatments.

## 4. Discussion

The present study suggests that, for the specific tea genotype and greenhouse conditions evaluated here, intermediate IBA concentrations (4000–6000 ppm) provided favorable conditions for adventitious root formation and subsequent growth in tea cuttings. While higher IBA doses enhanced root number and length, excessive concentrations did not further improve overall growth performance, indicating the presence of an optimal auxin threshold. This response pattern is consistent with the nonlinear nature of auxin-mediated adventitious rooting, in which insufficient auxin may fail to stimulate root initiation, whereas excessive auxin can reduce rooting efficiency or impair subsequent growth [28,29,30,31]. Therefore, the relatively better performance of the intermediate IBA range observed in the present study may reflect not only a rooting response but also a balance between root initiation, root system development, and shoot growth [30,32].

Indeed, many researchers [5,7,33,34] have reported that, similar to our study, the longest root development in tea cuttings in perlite rooting medium was obtained from tea cuttings treated with 6000 ppm IBA. However, Aydın and Kalkışım (2024) reported in their study that the best root development in hardwood tea cuttings was achieved with the application of 3000 ppm IBA [8]. Gonbad and Chokami (2009) stated that the most successful result in the rooting of Clone-100 tea cuttings was obtained from a combination of 2000 ppm NAA + IBA [35]. Rout (2006) found that the best root development in *Camellia sinensis* var. TV-20 tea cuttings was achieved with 75 ppm IBA [36]. In terms of rooting performance, previous tea propagation studies have reported considerable variability in rooting responses depending on cutting type, genotype, auxin concentration, pH, rooting medium, and nursery conditions. Hoque [33] reported that 4000 ppm IBA produced the highest rooting rate (79.85%), survival rate (57.60%), and root length (4.85 cm), whereas 6000 ppm IBA produced the highest root number (8.33 roots per cutting) in tea stem cuttings. Zenginbal et al. [6] found that 6000 ppm IBA combined with full-leaf cuttings resulted in 78.3% rooting, 90.0% survival, 14.77 cm root length, and 6.37 roots per cutting in the Turkish tea clone ‘Fener-3’. Similarly, Zenginbal et al. [5] reported rooting rates up to 85.0% and survival rates up to 91.7% in semi-hardwood tea cuttings, with the best rooting quality generally obtained from full-leaf cuttings treated with 4000–6000 ppm IBA. Yavaşı and Özcan [7] reported that 6000 ppm IBA resulted in 65.26% rooting, 4.90 cm root length, and 4.97 roots per cutting, while the pH 5.5 rooting medium produced 69.69% rooting and 9.41 cm root length. Vidanapathirana et al. [37] reported that an IBA-based rooting hormone produced 84.5% rooting at the 6th week and 55.8% rooting with 72.3% sprouting at the 4th week, outperforming several natural rooting agents. Compared with these studies, the present results are consistent with previous reports showing positive responses to IBA in tea cutting propagation and further indicate that, under the present greenhouse conditions, the most favorable response occurred within the intermediate range of 5000–6000 ppm IBA in perlite. Therefore, when comparing tea propagation studies, it should be considered that some studies define treatment success mainly by rooting percentage, whereas others emphasize root length, root number, sprouting, survival, or subsequent plantlet growth; consequently, the “optimal” IBA dose may vary according to the primary propagation criterion. Such variation among studies suggests that the optimum IBA dose should not be considered a fixed value for tea cuttings, but rather a treatment range that changes according to genotype, cutting type, physiological maturity, substrate conditions, and the rooting environment [30,38,39]. In this context, the 4000–6000 ppm range observed in the present study appears to represent an effective auxin window for the specific tea genotype evaluated here, under second-flush semi-hardwood cutting and perlite-based greenhouse conditions [38,39].

Variability among studies regarding the effect of IBA doses on rooting success in tea cuttings is expected because rooting responses are influenced by multiple biological and environmental factors. This can be attributed to factors such as the clonal structure and cultivar differences in the garden from which the cuttings are taken [40,41,42], the age of the plant and the time of sampling [9,43], cutting position [42,44,45], cutting length and thickness, the number and area of leaves on the cutting [46,47], endogenous hormones [48], rooting medium [47,49,50,51], pH [8], growth regulators [6,7,34,37,47], and cultural practices.

In addition to these factors, the physical and chemical characteristics of the rooting substrate may strongly influence auxin uptake, water availability, aeration, and root elongation, thereby modifying the apparent effectiveness of a given IBA dose [52,53,54]. Previous studies on woody and grafted cuttings have shown that rooting media differ in their capacity to retain water, provide aeration, and support callus and root development; therefore, differences in rooting medium can partly explain why similar auxin treatments produce different rooting responses across studies [52,54].

In the present study, vegetative tea cuttings pre-treated with *Aloe vera* showed greater root length and root number in clay soil than in perlite. This result indicates that *Aloe vera* gel may interact with substrate properties, particularly moisture retention and organic/mineral conditions, which can influence root elongation and early root establishment [52,55,56]. Similar observations in other cutting-propagated species suggest that the effectiveness of natural rooting stimulants is not independent of the propagation medium, since topsoil or organic substrates may provide a more favorable environment for the expression of biostimulant effects than inert substrates alone [55,56]. In contrast, cuttings rooted in perlite showed greater shoot height growth than those rooted in clay soil.

The greater shoot growth observed in perlite may be associated with its favorable aeration and drainage characteristics, which can reduce waterlogging around the cutting base and support early aboveground development after root initiation [52,54]. However, the contrasting response between root elongation in clay soil and shoot growth in perlite also indicates that root and shoot traits may respond differently to the same propagation environment [32,57].

This indicates that *Aloe vera* application may be more effective in promoting root elongation under soil-based rooting conditions, whereas perlite appears to support better shoot elongation. These findings suggest that the observed response to *Aloe vera* gel may be partly associated with bioactive compounds reported in the literature, such as gibberellins and salicylic acid; however, the chemical composition of the *Aloe vera* gel used in the present study was not analytically characterized [58].

*Aloe vera* gel has also been reported to contain several bioactive constituents, including polysaccharides, amino acids, vitamins, gibberellins, and salicylic acid, which may contribute to root initiation, root elongation, and early bud development in stem cuttings [59,60,61,62,63]. In experimental studies on different cutting-propagated species, *Aloe vera* treatments have improved rooting percentage, root length, or rooting speed, supporting its potential role as a natural rooting biostimulant [16,56,62]. For tea specifically, *Aloe vera* gel and other natural rooting substances have been evaluated as alternatives to synthetic rooting hormones, especially in propagation systems where organic or low-input nursery practices are desired [37].

Overall, the findings of the present study should be interpreted through the combined effects of auxin dose, rooting medium, and root–shoot balance. The 5000 ppm IBA treatment provided the highest rooting and survival performance, whereas 6000 ppm IBA increased root number, suggesting that maximum rooting percentage and maximum root proliferation do not necessarily occur at the same auxin level [30,32]. From a nursery-production perspective, this distinction is important because a successful tea plantlet produced from cuttings requires not only root initiation but also a functional balance between root development, shoot growth, and post-transplant establishment [57,64]. Thus, under the tested greenhouse conditions, intermediate IBA doses in perlite appear suitable for producing rooted tea cuttings with favorable early growth responses, while *Aloe vera* gel may be considered a potential natural alternative particularly for soil-based or organic propagation systems [37,55,56].

## 5. Conclusions

This study showed that rooting performance and early growth of vegetative tea (*Camellia sinensis*) cuttings were influenced by the auxin concentration and rooting medium under the tested greenhouse conditions. Among the evaluated treatments, intermediate IBA doses (4000–6000 ppm) applied in perlite provided the most favorable balance between rooting success, survival rates, and subsequent shoot growth. The combination of 5000 ppm IBA with perlite resulted in the highest rooting and survival percentages, while 6000 ppm IBA promoted the greatest root number.

The rooting medium appeared to influence root–shoot allocation, with clay soil favoring root elongation and perlite supporting early shoot growth under the tested conditions. *Aloe vera* gel was associated with greater root length particularly in soil-based media, suggesting its potential as a natural rooting stimulant for tea propagation systems relying on organic substrates.

Overall, the findings indicate that, under the tested conditions, intermediate IBA application in perlite may offer an effective approach for nursery-scale propagation of tea cuttings, while *Aloe vera* gel may serve as a potential sustainable alternative under soil-based conditions. However, the present study should be interpreted as an applied propagation experiment rather than a physiological, biochemical, or molecular study. Therefore, future research should focus on evaluating IBA applications in soil substrates to improve the transferability of nursery protocols to field conditions and employ full factorial designs including all IBA concentrations and *Aloe vera* gel treatments across different rooting media to better clarify treatment × substrate interactions. In addition, future studies should compare different immersion durations for both IBA solutions and *Aloe vera* gel and integrate physiological, biochemical, and molecular analyses, such as photosynthetic pigment concentrations, antioxidant enzyme activities, endogenous hormone levels, and rooting-related gene expression, to better clarify the mechanisms underlying treatment-induced rooting responses.

## Figures and Tables

**Table 1:** Experimental design of rooting treatments applied to vegetative tea cuttings.

Trial No	Rooting and Growth Environment	IBA and *Aloe vera* Doses	Number of Cuttings in Repetitions		
			**R1**	**R2**	**R3**
T1	Perlite (100%)	Control (none)	10	10	10
T2	Clay Soil (100%)	Control (none)	10	10	10
T3	Perlite (100%)	2000 ppm IBA	10	10	10
T4	Perlite (100%)	3000 ppm IBA	10	10	10
T5	Perlite (100%)	4000 ppm IBA	10	10	10
T6	Perlite (100%)	5000 ppm IBA	10	10	10
T7	Perlite (100%)	6000 ppm IBA	10	10	10
T8	Perlite (100%)	7000 ppm IBA	10	10	10
T9	Perlite (100%)	8000 ppm IBA	10	10	10
T10	Perlite (100%)	*Aloe vera* gel	10	10	10
T11	Clay Soil (100%)	*Aloe vera* gel	10	10	10

**Table 2:** Measured plant parameters.

Measured Parameters	Measurement Method
Plantlet height	Measured with a ruler.
Root Collar Diameter	Measured with a digital caliper (Insize, İstanbul, Türkiye)
Root Number	Determined by counting on a millimeter paper template.
Biomass (Belowground and aboveground)	3 plants were randomly uprooted from each trial area, washed, cut from the root collar diameter and separated into above ground and underground. Then, their weights were determined by weighing.
Root Length	Measured with a ruler on millimeter paper
Leaf Number	Determined by counting the leaves on the plant.
Flower Bud Number	Determined by counting visible flower buds or flowers on each cutting.

**Table 3:** Effects of Rooting Medium and Pre-treatment on Root and Cutting Parameters of Vegetative Tea Cuttings (Two-way ANOVA).

Rooting Medium	Pre-Treatment	Root Length (mm)		Number of Roots (Per Cutting)		Cutting Length (mm)	
		Mean	SD	Mean	SD	Mean	SD
Perlite	Untreated	24.69	15.16	20.31	16.42	121.46	21.11
	*Aloe vera* gel	33.58	23.88	25.45	21.92	112.82	19.50
	Average	29.63	20.69	23.17	19.57	116.66	20.41
Clay Soil	Untreated	72.88	36.68	24.50	18.72	92.07	17.87
	*Aloe vera* gel	75.97	28.89	29.79	13.98	96.45	14.21
	Average	74.56	32.22	27.37	16.28	94.45	15.90
Perlite + Clay Soil	Untreated	48.78	36.90	22.41	17.45	106.77	24.35
	*Aloe vera* gel	54.23	33.78	27.56	18.37	104.84	18.83
ANOVA Results (F values and significance levels)							
Factor		Root Length (mm)		Number of Roots		Cutting Length (mm)	
Rooting Medium		48.792 (*p* < 0.001)		0.975 (*p* = 0.327)		27.585 (*p* < 0.001)	
Pre-treatment		0.853 (*p* = 0.359)		1.458 (*p* = 0.231)		0.240 (*p* = 0.626)	
Rooting Medium × Pre-treatment		0.200 (*p* = 0.656)		0.001 (*p* = 0.986)		2.237 (*p* = 0.139)	

**Table 4:** Analysis results showing the survival rate, rooting rate, and leaf emergence rate of cuttings after 120 days under different treatment combinations.

Treatments	Survival Rate of Cuttings (%)			Rooting Rate of Cuttings (%)			Budding Leaves (%)		
	Mean	SD	Grouping	Mean	SD	Grouping	Mean	SD	Grouping
Perlite (Control)	0.60	0.17	b	0.60	0.17	cd	0.36	0.19	c
Soil (Control)	0.60	0.20	b	0.57	0.15	d	0.53	0.17	abc
2000 ppm IBA + perlite	0.83	0.15	ab	0.73	0.12	bcd	0.52	0.23	abc
3000 ppm IBA + perlite	0.90	0.10	ab	0.77	0.25	bcd	0.64	0.05	abc
4000 ppm IBA + perlite	0.87	0.15	ab	0.87	0.15	abc	0.53	0.09	abc
5000 ppm IBA + perlite	1.00	0.00	a	1.00	0.00	a	0.77	0.15	a
6000 ppm IBA + perlite	0.90	0.10	ab	0.90	0.10	ab	0.59	0.09	abc
7000 ppm IBA + perlite	0.77	0.06	b	0.77	0.06	bcd	0.48	0.25	abc
8000 ppm IBA + perlite	0.73	0.21	b	0.90	0.10	ab	0.60	0.09	abc
*Aloe vera* + perlite	0.70	0.10	b	0.67	0.15	bcd	0.37	0.04	c
*Aloe vera* + soil	0.67	0.25	b	0.67	0.15	bcd	0.75	0.04	ab
F	2.873			3.302			2.474		
*p*	0.019			0.009			0.037		

Means followed by different lowercase letters (a–d) within the same column are significantly different according to Duncan’s Multiple Range Test (*p* < 0.05). Means sharing at least one common letter are not significantly different.

**Table 5:** One-way ANOVA results showing the effects of different pretreatments and growth media on root length, number of roots, and cutting length (Control: Cuttings not treated with *Aloe vera* or IBA (Indole-3-butyric acid); different letters within a column indicate significant differences according to Duncan’s test (*p* < 0.05)).

Treatment	Root Length (mm)			Number of Roots (Roots per Cutting)			Vegetative Cutting Length (mm)		
	Mean	S.D.	Grouping	Mean	S.D.	Grouping	Mean	S.D.	Grouping
Perlit (Control)	24.69	15.17	e	20.31	16.42	cde	121.46	21.11	bc
Soil (Control)	72.88	36.68	ab	24.50	18.72	bcde	92.07	17.87	f
2000 ppm IBA + perlite	40.70	29.35	cde	13.61	10.71	e	149.46	15.30	a
3000 ppm IBA + perlite	31.85	29.68	de	17.23	13.82	de	147.71	25.02	a
4000 ppm IBA + perlite	45.64	26.38	cd	24.54	16.87	bcde	127.34	13.86	bc
5000 ppm IBA + perlite	45.54	30.44	cd	23.11	17.37	bcde	122.09	21.18	bc
6000 ppm IBA + perlite	56.48	26.28	bc	40.62	19.38	a	114.27	22.04	cd
7000 ppm IBA + perlite	46.05	30.03	cd	34.05	18.54	ab	106.20	10.68	de
8000 ppm IBA + perlite	43.40	24.28	cde	27.92	13.04	bcd	110.57	21.59	cd
5 s *Aloe vera* + perlite	33.58	23.88	de	25.45	21.92	bcde	112.82	19.50	cd
5 s *Aloe vera* + soil	75.97	28.89	a	29.79	13.98	abc	96.45	14.21	ef
F	6.045			4.484			17.867		
*p*	0.000			0.000			0.000		

**Table 6:** One-way ANOVA results for shoot height growth up to 15 weeks after planting of rooted cuttings under different treatment combinations.

Plantlet Height Growth (mm) by Weeks		Perlite (Control)	Soil (Control)	2000 ppm IBA + Perlite	3000 ppm IBA + Perlite	4000 ppm IBA + Perlite	5000 ppm IBA+ Perlite	6000 ppm IBA + Perlite	7000 ppm IBA + Perlite	8000 ppm IBA + Perlite	*Aloe vera* + Perlite	*Aloe vera* + Soil
Week-3	Mean	2.88	1.75	0.14	0.43	2.29	2.88	2.12	1.63	1.75	2.62	1.25
	SD	1.808	1.282	1.069	1.988	2.059	1.642	1.727	0.744	0.886	1.408	0.707
		a	ab	b	b	a	a	a	ab	ab	a	ab
	F (p)	2.900 (0.004)										
Week-5	Mean	3.75	3.5	0.57	2.14	4.71	3.87	3.88	2.5	2.75	3	2.63
	SD	1.669	1.773	1.988	1.464	4.889	1.458	1.727	0.926	1.581	1.852	0.744
		ab	ab	c	bc	a	ab	ab	abc	abc	ab	abc
	F (p)	2.138 (0.032)										
Week-7	Mean	4.75	4.75	1.57	4.29	10.86	5.25	6.13	3.5	4.13	5	4
	SD	1.282	1.909	3.101	2.563	6.283	1.282	2.532	1.512	1.458	1.512	1.069
		b	b	c	bc	a	b	b	bc	bc	b	bc
	F (p)	5.662 (<0.001)										
Week-9	Mean	5.63	6.13	3	5.14	10.29	6.38	8.25	4.75	4.62	6.13	5.37
	SD	1.598	2.532	2.16	1.773	6.55	1.598	3.151	1.982	1.302	1.246	0.744
		bcd	bc	d	cd	a	bc	ab	cd	cd	bc	bcd
	F (p)	3.934 (<0.001)										
Week-11	Mean	6.25	7	3.57	5.57	12.71	7	8.63	6.25	6.5	7.5	6.75
	SD	1.669	2.828	1.988	2.992	7.477	1.309	2.875	2.375	2.449	1.927	0.707
		bc	bc	c	bc	a	bc	b	bc	bc	b	bc
	F (p)	3.975 (<0.001)										
Week-13	Mean	8.38	9	3.86	7.43	12.43	9.25	11.5	7.63	9.88	9.88	8.87
	SD	1.768	2.507	1.952	3.735	7.764	1.282	3.251	2.925	2.997	3.357	1.458
		bc	abc	d	c	a	abc	ab	bc	abc	abc	abc
	F (p)	3.236 (0.002)										
Week-15	Mean	9.13	11.25	4.43	8.29	13.71	10.75	12.88	7.88	12.25	12	10.88
	SD	1.727	2.252	2.07	3.45	9.232	0.707	3.044	1.808	3.105	4.84	1.959
		bcd	abcd	e	cde	a	abcd	ab	de	abc	abcd	abcd
	F (p)	3.845 (<0.001)										

Different letters within a row indicate significant differences according to Duncan’s test (*p* < 0.05).

## Data Availability

The original contributions presented in the study are included in the article. Further inquiries can be directed to the corresponding author.

## Abbreviations

The following abbreviations are used in this manuscript:

IBA	Indole-3-butyric acid
ANOVA	Analysis of variance
RCBD	Randomized complete block design
SPSS	Statistical Package for the Social Sciences
SD	Standard deviation
ppm	Parts per million
mm	Millimeter
cm	Centimeter
m	Meter
kg/da	Kilogram per decare
NPK	Nitrogen–phosphorus–potassium compound fertilizer
FAO	Food and Agriculture Organization of the United Nations
TÜBİTAK	The Scientific and Technological Research Council of Türkiye
USD	United States dollar
p	Probability value/significance level
F	F-statistic value
R1, R2, R3	Replication 1, Replication 2, Replication 3
T1–T10	Treatment groups
P	Perlite
sec	Second
